# Posterior cingulate cortex can be a regulatory modulator of the default mode network in task-negative state

**DOI:** 10.1038/s41598-019-43885-1

**Published:** 2019-05-20

**Authors:** Regina W. Y. Wang, Wei-Li Chang, Shang-Wen Chuang, I-Ning Liu

**Affiliations:** 1 Design Perceptual Awareness Lab (D:PAL), National Taiwan University of Science and Technology (Taiwan Tech), Taipei, Taiwan; 2 The Department of Design, National Taiwan University of Science and Technology (Taiwan Tech), Taipei, Taiwan; 3 Taiwan Building Technology Center, National Taiwan University of Science and Technology (Taiwan Tech), Taipei, Taiwan

**Keywords:** Personality, Computational neuroscience, Social behaviour

## Abstract

In recent years, the regulation of brain networks and interactions between different brain regions have become important issues in neuroscience. Effective connectivity can be employed to understand the modulatory mechanisms of brain networks. Previous studies have used the task-positive mode to examine effective connectivity between brain regions and very few studies have considered the task-negative mode to explore effective connectivity using electroencephalography (EEG). In the present study, high-density EEG experiments were conducted in 85 participants to measure EEG effective connectivity in relevant default mode network (DMN) brain regions (i.e., the medial prefrontal cortex [mPFC], posterior cingulate cortex [PCC], precuneus, and right frontal and left occipital regions) to observe the effects of different task-negative modes (eyes-open/eyes-closed state) and personality traits (introversion/extroversion). The results showed that in the eyes-closed state, the PCC had significantly increased effective connectivity and played a prominent role as a regulatory modulator of outflow to other regions mediated by alpha rhythms. The mPFC was a regulatory modulator of outflow in the eyes-open state mediated by delta rhythms. The introvert group showed stronger co-modulations in the relevant DMN regions than the extrovert group.

## Introduction

Communication and coordination within a brain network to recruit different brain regions is achieved through the neuro-modulation of external signals^1^. Friston proposed the concept of effective connectivity to describe the causal relationship existing between the neuronal activity patterns observed in the neurons, nerve plexuses, and brain regions^2^. This construct is used to describe the interaction between brain regions occurring within brain networks.

Among neuroimaging techniques, electroencephalography (EEG) has a higher time resolution that enables the correlation of real time brain dynamic changes with behavioral responses at the scale of microseconds to minutes. Using a 60-channel transcranial magnetic stimulation-compatible EEG, Massimini observed a disruption of the cortical effective connectivity when consciousness fades during sleep^3^. The EEG responses during the rapid eye movement sleep episode were similar to those observed in wakefulness and the brain neuro-modulation was observed in different states^4^. Many studies have found that EEG effective connectivity reflected the brain’s immediate activation during tasks and physiological states such as sleep^5^, memory^6^, and motor-related tasks^7^. Raichle found that brain regions in the default mode network (DMN) are nevertheless activated in the absence of tasks^8^. Several lines of evidence suggest that the brain regions underlying the DMN are even activated in a task-negative mode when the eyes are closed. It has been reported that having eyes closed or eyes open affects the activation level of DMN regions^9^. The power of the middle frequency bands was significantly reduced over the posterior areas and the low frequency band power increased in the forebrain area in the DMN from the eyes-closed to eyes-open state^10^.

Cognitive processes including mind wandering^11^, self-reference^12^, and social cognition^13^ enhance the activity of DMN regions recorded using EEG. Personality psychology aims to understand stable differences between individuals that may affect decision-making behavior^14^. EEGs exhibit electrical oscillations in the brain that are important links between information processing and cognition^15^. Introversion and extroversion are the most important constructs in personality trait theory. Jung believed that introverts focus on inner psychological activity and extroverts tend to focus on the external world^16^. It has been reported that eyes-closed or eyes-open states led to different levels of arousal in introvert and extrovert participants, resulting in different brain activation^17^. Previous studies have shown that the difference between introversion and extroversion is reflected in the low frequency spectral power measured in the forebrain and hindbrain^18^. It is proposed in this study that introversion and extroversion lead to different levels of arousal affecting EEG effective connectivity occurring during DMN brain region activation.

The DMN provides an innovative point of view to explore the operating mechanisms of the brain^19^. The brain regions of the DMN not only provide a cognitive and physiological neurobiological system necessary for the brain to respond to external stimuli^20^, but also play an important role in social cognition^13^ such as mediating the ability to infer the thoughts and feelings of other people^21^. The DMN involves the dynamic activation of different brain regions^22,23^. Repeated observation has shown that the medial prefrontal cortex (mPFC), posterior cingulate cortex (PCC), and precuneus exhibit high levels of activity during task-negative (resting) states and reduced activity during external task-positive states resulting in the characterization of these regions as belonging to a DMN^8,19,24–28^. Resting increases the connectivity between the precuneus and DMN^29,30^. To identify functional hubs, we first calculated connection strength values for all nodes (i.e., dipole locations in the present study) in the relevant network regions^31^. The DMN regions of interest (i.e., mPFC, PCC, and precuneus) were based on the equivalent dipole location analysis of 5610 ICs (Talairach xyz coordinate values)^32,33^ collected from the present study, corresponding to the defined Brodmann areas (for details see Materials and Methods, Measure of effective connectivity). The anterior hub of the DMN is the mPFC^23,29,34,35^, which is associated with personality expression^36^, decision making^37^, thinking^20^, and other high-level cognitive functions. The posterior hub of the DMN is the PCC^23,29,34,35^, which is associated with emotion^38^, cognition^39^, awareness^40^, and arousal^39^. The precuneus is located above and posterior to the PCC, and is involved in self-consciousness^41^, memory^42^, and visuospatial^43^ function. Several studies have found that meditation, sleep deprivation, and drug abuse can alter both the pattern of the brain regions comprising the DMN and the synchronized oscillations between these brain regions^28^. Alterations of the DMN are more likely to lead to Alzheimer’s disease^20^, schizophrenia, autism, and other mental disorders.

**Figure 1:**
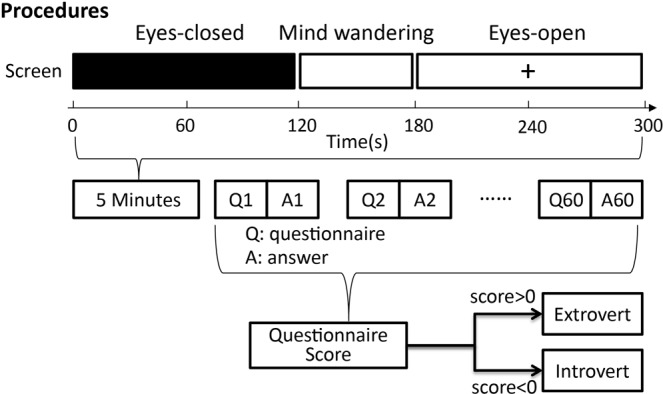
The procedure of the experiment. At the beginning of the experiment, 85 subjects remained in the eyes-closed state for two minutes, mind-wandering for one minute, and eyes-open for two minutes. Then, the subjects answered 60 questions from the personality questionnaire. According to the questionnaire results, subjects with a total score greater than 0 were classified as extroverts, while those with a total score less than 0 classified as introverts.

The study was designed as a two-factor experiment in which the interaction of one independent variable (i.e., visual inspection, eyes-open/eyes-closed) with another independent variable (i.e., personality trait - introversion/extroversion) was analyzed by measuring EEG effective connectivity between brain regions of the DMN. The goals of the study were to investigate if there was any change in EEG effective connectivity in DMN regions with different task-negative conditions (i.e., eyes-open/eyes-closed) and personality traits (i.e., introversion/extroversion). The experimental procedure is shown in Fig. 1.

## Results

The results are presented in four sections: (a) behavioral results of the personality classification; (b) EEG brain regions and frequency band oscillations in different task-negative conditions and with different personality traits; (c) effective connectivity in task-negative conditions; and (d) effective connectivity across introversion and extroversion traits.

### Behavior results of the personality classification

In the procedure of the experiment (Fig. 1), the introversion/extroversion of the 85 subjects was determined using the MBTI personality questionnaire, dividing the subjects into 43 introverts and 42 extroverts.

### EEG brain regions and frequency band oscillations in task-negative modes and individuals with different personality traits

**Figure 2:**
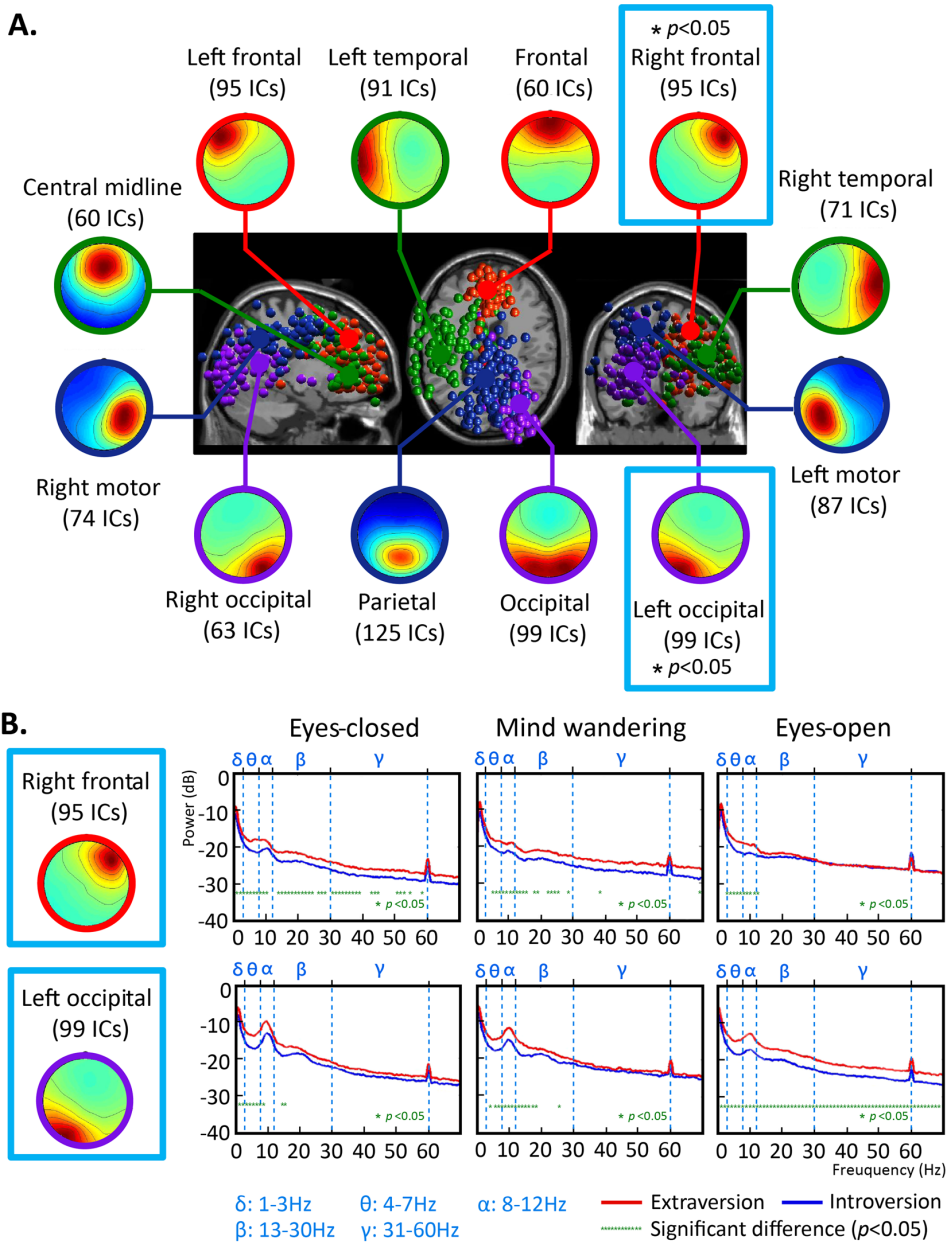
Result of ICA clustering and spectral analysis across subjects. (**A**) Presents a diagram of the scalp and the dipole location from ICA clustering across 85 subjects. According to 12 common brain regions, equivalent dipoles are divided into Frontal (red), Central (green), Parietal (blue), and Occipital (purple). (**B**) Shows that in the right frontal and left occipital regions, the spectral power is significantly different (Wilcoxon rank-sum test, p < 0.05) between introverts and extroverts, in the task-negative conditions of eyes-closed, mind wandering, and eyes-open. In the right frontal region, the frequency band powers with significant differences between introverts and extroverts are as follows. During the eyes-closed, significantly different delta band (1–3 Hz), theta band (4–7 Hz), alpha band (8–12 Hz), beta band (13–30 Hz), and low gamma band (31–40 Hz) power were found; during mind-wandering, the theta (4–7 Hz), alpha (8–12 Hz), and low beta band (13–24 Hz) powers are significantly different; during eyes-open, there are significant differences in the theta band (4–7 Hz) and alpha band (8–12 Hz). The frequency band powers that have significant difference in the left occipital region are as follows. During the eyes-closed state, significantly different delta band (2–3 Hz), theta band (4–7 Hz), and alpha band (8–12 Hz) power were found; during mind-wandering: theta (4–7 Hz), alpha (8–12 Hz), and low beta band (13–19 Hz) are significantly different; during the eyes-open state, there are significant differences across a wide frequency band (1–70 Hz). *Significant difference between introversion/extroversion (Wilcoxon rank-sum test, p < 0.05).

The data from the right frontal region and left occipital region were compared for statistical differences, finding significant differences across different brain regions and frequencies with different task-negative conditions and personality traits (Wilcoxon rank-sum test, p < 0.05; Fig. 2A,B). In the right frontal region, the theta band (4–7 Hz) and alpha band (8–12 Hz) powers were significantly different between introverts and extroverts and between the different task-negative states. In the left occipital region, the delta (2–3 Hz), theta (4–7 Hz), and alpha band (8–12 Hz) powers were significantly different between introverts and extroverts and between the different task-negative states. In either the right frontal region or left occipital region, the alpha band (8–12 Hz) powers was apparently significant difference between introverts and extroverts interacted with different task-negative states. For details see Figure legends.

Both the right frontal region and left occipital region, and the DMN regions of interest, i.e., the mPFC, PCC, and precuneus, were therefore investigated to find the EEG effective connectivity following independent component analysis (ICA) of the findings presented in Fig. 2. For details see ICA and clustering.

### Effective connectivity in task-negative conditions

**Figure 3:**
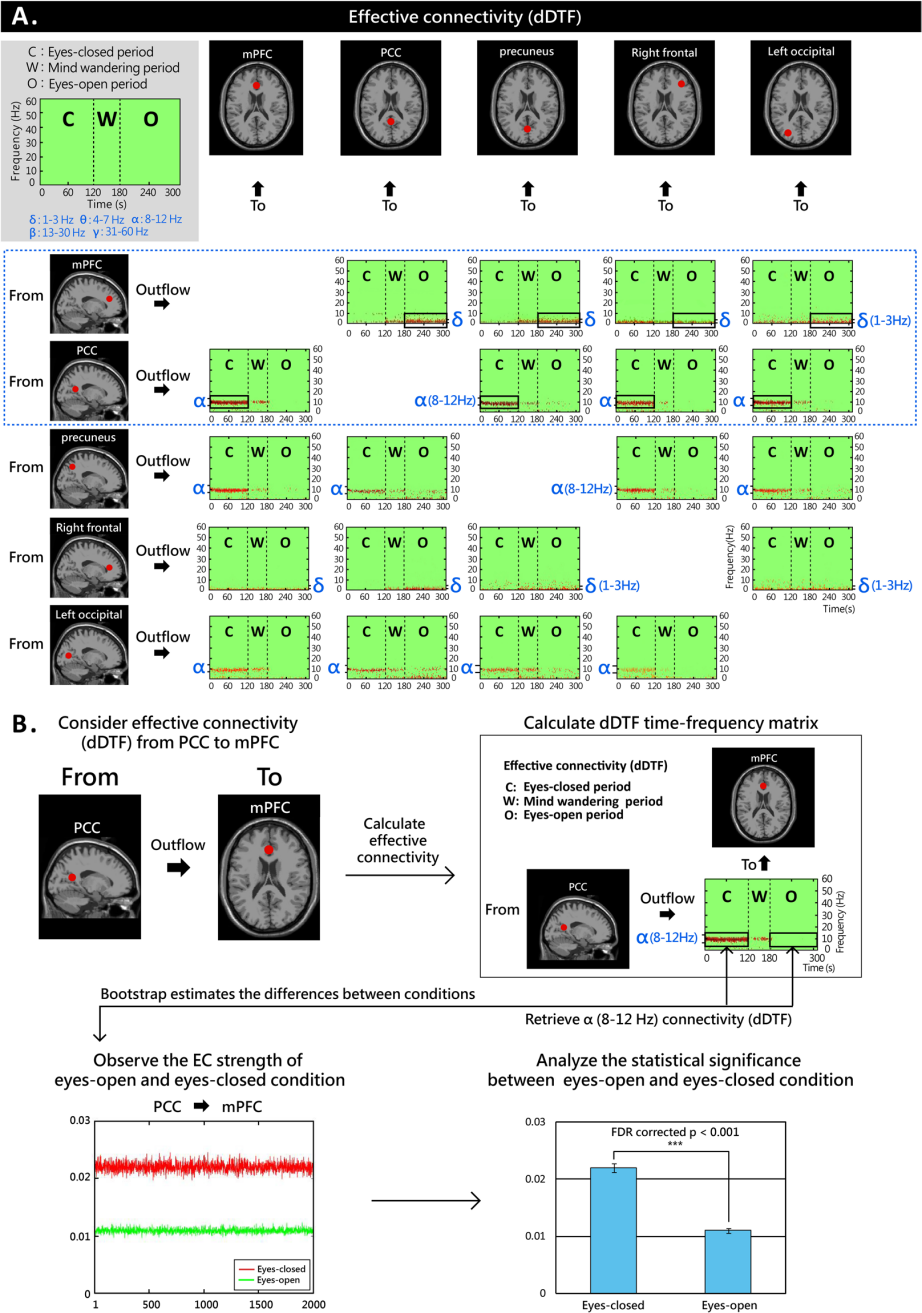
Effective connectivity analysis on data from 85 subjects. Analysis of the connection strength values (dDTF) between DMN-relevant brain regions (mPFC, PCC, precuneus, and right frontal and left occipital regions) with respect to the effects of different task-negative conditions identify the PCC and mPFC as functional hubs. During the eyes-closed state, the PCC regulates a majority of the other four brain regions (PCC → mPFC, PCC → precuneus, PCC → right frontal region, PCC → left occipital region) through the alpha band (8–12 Hz). The direct Directed Transfer Function (dDTF) value of significant effective connectivity is represented by red dots; the higher the density of red dot clusters in the frequency band areas, the stronger the effective connectivity would be (**A**). The Source Information Flow Toolbox (SIFT)^44^ of MATLAB was used for the analysis of effective connectivity (i.e., the causal relationship between activity measured in different brain regions). The dDTF^45,46^ was used to calculate the causal relationship between brain regions by calculating the intensity values of effective connectivity between brain regions at different times and EEG frequencies. The dDTF values of the time-frequency matrices corresponding to the brain regions were extracted from a total of 85 dDTF, summed, and averaged to obtain the dDTF time frequency matrix (5 × 5) of the causal relationships of the five brain regions. Bootstrap statistics were used by EEGLAB to assess the differences between conditions (p < 0.05). Its processing flow is shown in (**B**). For specifications about the measurement of the dDTF and information flow analysis see the manuscript (pp. 25–30). During the eyes-open state, the mPFC regulates a majority of the four other brain regions through the delta band (1–3 Hz), and the effective connectivity is significant. A 5 × 5 dDTF time-frequency grid was used to show that the forebrain regions (mPFC, right frontal region) affect the hind brain regions (PCC, precuneus, left occipital region) during the eyes-open state. On the contrary, the hindbrain regions (PCC, precuneus, left occipital region) affect the forebrain regions (mPFC, right frontal region) during the eyes-closed state (Bootstrap resampling, p < 0.05).

Figure 3A shows the EEG effective connectivity for the five explored brain regions (mPFC, PCC, precuneus, right frontal region, and left occipital region). The DMN regions of interest (mPFC, PCC, precuneus, right frontal region, and left occipital region) as defined by previous studies^8,19,24–30^ were based on the equivalent dipole location analysis of 5610 ICs (Talairach xyz coordinate values)^32,33^ collected in the present study. The study detected 273 components in the mPFC, 81 components in the PCC, 74 components in the precuneus, 95 components in the right frontal region, and 99 components in the left occipital regions.

To identify the functional hubs of the mPFC and PCC referring to previous studies^23,29,34,35^, we calculated connection strength values for all nodes (dipole locations in the present study) in the relevant network regions^31^ of the mPFC, PCC, precuneus, left occipital region, and right frontal region. Figure 3A shows that during the eyes-closed state (the area labeled “C” on Fig. 3A), effective connectivity was mostly found in the alpha band. The PCC can strongly regulate a majority of the other four brain regions (i.e., PCC outflow to the mPFC, precuneus, right frontal region, and left occipital region) through the alpha band (8–12 Hz), whereas the precuneus regulates the other regions with lower effective connectivity values through the alpha band. In Fig. 3A, significant effective connectivity is represented by the red dots for the direct Directed Transfer Function (dDTF) value, with higher densities of red dot clusters in the frequency band areas reflecting stronger effective connectivity. The Source Information Flow Toolbox (SIFT)^44^ in MATLAB was used for the analysis of effective connectivity (i.e. the causal relationship between activity measured in different brain regions). The dDTF^45,46^ was used to calculate the causal relationship between brain regions by calculating the intensity values of effective connectivity between brain regions at different times and EEG frequencies (for details see Measure of Effect Connectivity, Information Flow Analysis).

Effective connectivity was mostly found in the delta band in the eyes-open state (the area labeled “O” on Fig. 3A). Furthermore, the mPFC strongly regulated a majority of the other four brain regions (i.e., mPFC outflow to the PCC, precuneus, right frontal region, and left occipital region) through the delta band (1–3 Hz). However, compared to the eyes-closed and eyes-open state, the mind wandering state (the area labeled “W” on Fig. 3A) was found to have the least power for all frequency bands in all regions. The effective connectivity between different regions was the least significant value of the dDTF as represented by the red dots, with lowest density red dot clusters in the frequency band areas representing weaker effective connectivity.

**Figure 4:**
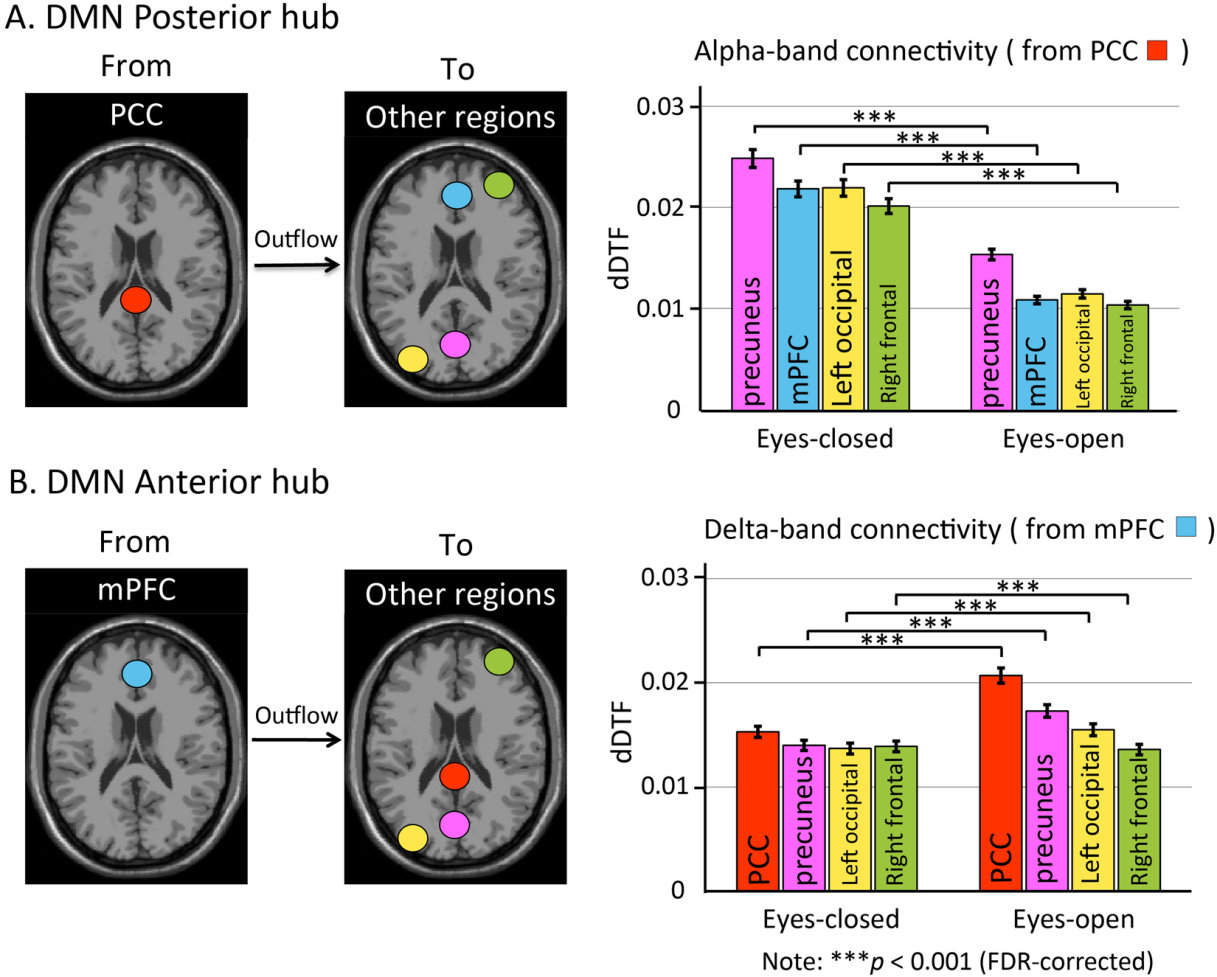
Effect of task-negative conditions (eyes-open/eyes-closed) on the effective connectivity of the DMN anterior (mPFC) and posterior (PCC) hubs. The results show that the EEG effective connectivity through the alpha band of the regulatory PCC outflow to the other four brain regions (precuneus, mPFC, left occipital region, and right frontal region) is significantly different between eyes-closed and eyes-open conditions. The effective connectivity in eyes-closed conditions is significantly greater than that in eye-open conditions (two sample t-test, p < 0.001; **A**). The EEG effective connectivity through the delta band of the regulatory mPFC outflow to the other four regions (PCC, precuneus, left occipital region, and right frontal region) is significantly different between eyes-open and eyes-closed conditions. The effective connectivity in the eyes-open condition (with the PCC, precuneus, and left occipital region) is significantly greater than that in the eyes-closed condition (two sample t test, p < 0.001; **B**).

The functional hubs of the mPFC and PCC were identified from the effective connection strength values calculated in relevant network regions (Fig. 3A). The data suggest that in the eyes closed state the brain regions reciprocally regulate their activity in the alpha band, while they mostly operate in the delta band with eyes open. In particular, it was found that in the eyes-closed condition, the regulation was mainly occurring in the alpha band in the PCC. In the eyes-open condition, the regulation in the mPFC was mainly found in the delta band. Increases in effective connectivity with eyes-closed in PCC and eyes-open in mPFC were further observed (Fig. 4). A two-sample t-test was used to determine the whether there were significant differences between the experimental conditions. The effective connectivity through the alpha band from the regulatory PCC outflow to the precuneus, mPFC, right frontal region, and left occipital region was significantly different between the eyes-open and eyes-closed conditions (Fig. 4A), with connection values in the eyes-closed conditions significantly stronger than those in the eyes-opened conditions. The effective connectivity through the delta band from the mPFC outflow to the PCC, precuneus, left occipital region, and right frontal region was significantly different between the eyes-open and eyes-closed conditions (Fig. 4B), with connection values in the eyes-open significantly stronger than those in the eyes-closed conditions (two-sample t-test, p < 0.001, false discovery rate [FDR]-corrected).

### Effective connectivity of the introversion and extroversion traits

**Figure 5:**
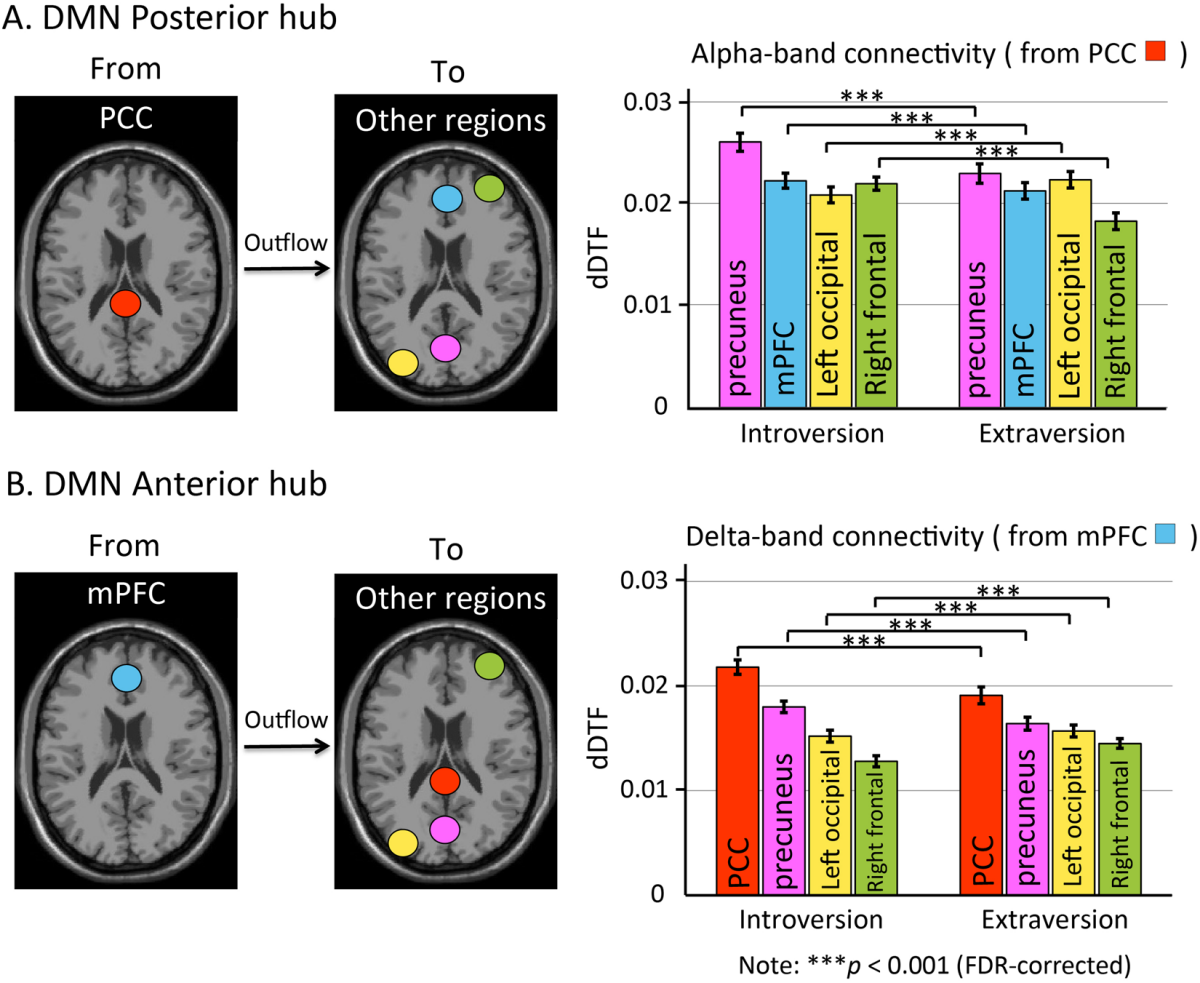
Effect of personality traits (introversion/extroversion) on the effective connectivity of DMN anterior (mPFC) and posterior (PCC) hubs. The strength of effective connectivity through the alpha band of the regulatory PCC outflow to the mPFC, precuneus, and right frontal region in introverts is significantly greater than the connectivity measured in extroverts (two-sample t-test, p < 0.001). On the contrary, the strength of effective connectivity from the PCC to the left occipital region is significantly lower than the connectivity measured in extroverts (**A**). The strength of effective connectivity through the delta band, from the regulatory mPFC outflow to the PCC and precuneus in the introvert group is significantly greater than the effective connectivity of the extrovert group. The strength of the effective connectivity from the mPFC to the left occipital and right frontal regions is significantly lower than the connectivity measured in the extroverts (**B**). The results show that in introverts, both the PCC and mPFC have strong modulatory effects on the other DMN brain regions and show a stronger effective connectivity than in extroverts (two-sample t-test, p < 0.001).

Effective connectivity in introverts and extroverts showed significant differences (Fig. 5). The effective connectivity from the PCC outflow to the mPFC, precuneus, right frontal region, and left occipital region through the alpha band was significantly different between the introvert and extrovert groups (Fig. 5A). The effective connectivity through the alpha band from the regulatory PCC outflow to the precuneus, mPFC, and right frontal region was significantly stronger in the introvert group than in the extrovert group. The effective connectivity from the mPFC to the PCC, precuneus, left occipital region, and right frontal region differed significantly between the introvert and extrovert groups (Fig. 5B). The effective connectivity through the delta band from the mPFC to the PCC and precuneus was stronger in introverts than in extroverts (two-sample t-test, p < 0.001, FDR-corrected).

## Discussion

The majority of the studies on the DMN have used functional magnetic resonance imaging (fMRI), a technique that cannot detect the roles played by different frequency bands in the DMN. The present study analyzed EEG effective connectivity to show that DMN brain regions use different frequency bands to regulate brain activity in different states (i.e., eyes-open vs eyes-closed). In particular, the alpha band (8–12 Hz) and delta band (1–3 Hz) were found to regulate brain activation in the eyes-open and eyes-closed states, respectively. We found that the PCC and mPFC were the main hubs modulating the other DMN brain regions in the study, as shown in Figs 3–5. There was increasing EEG effective connectivity in terms of major regulatory outflow in the DMN regions with effects depending on different task-negative conditions (i.e., eyes-open and eyes-closed) and personality traits (i.e., introversion and extroversion).

### The PCC is the major regulatory outflow to other regions during the eyes-closed state and the mPFC is the major regulatory outflow during the eye-opened state

Concerning the effect of a single factor (eyes-open/eyes-closed) on the DMN regions of interest (the PCC, mPFC, precuneus, left occipital region, right frontal region), in the present study, the eyes-closed states showed major outflow in neuro-modulatory mechanisms (Figs 3A and 4A). The present results suggest that the PCC plays an important role in task-negative modes and regulates the brain regions of the DMN through alpha rhythm modulation. Figure 3A shows the dDTF value of the effective connectivity represented by a red dot, where the density of red dot clusters in a frequency band area reflecting the strength of the effective connectivity. There was distinct regulation from the PCC outflow to the other DMN regions where the strength of the EEG effective connectivity through the alpha band is much greater than that of the other frequency bands. The previously reported data from the fMRI effective connectivity analysis suggest that the PCC in the eyes-closed state influences the activation of the mPFC^47^. fMRI has been used to investigate the role of the PCC in the modulation of the DMN activity. It has been found that the PCC plays a pivotal role in the functional connectivity of the DMN. The PCC has a strong interaction with the precuneus and direct connectivity with other DMN regions^48^, supporting the hypothesis put forward in the present study.

Recent findings have shown that the PCC plays an important role in the regulation of cognitive processes^49^ and that PCC and precuneus dysfunctional activation^50^ are associated with cognitive dysfunction^20^ in patients with Parkinson’s Disease. During the eyes-closed state, the cognitive load is reduced^51^ and the alpha rhythm of the hind brain increases rapidly in the pre-sleeping stage^52^. Ben-Simon *et al*.^53^ used an fMRI approach to show that the eyes-closed state triggers alpha modulation (a correlate of cortical idling)^54^. These previous studies together with the present results suggest that in the eyes-closed state the PCC is the main hub with the strongest effective connectivity in the regulation of brain activity, such as outflow, and uses the alpha band to modulate relevant brain regions.

In the eyes-open state, the mPFC plays a major role in the regulation of the other brain regions (i.e., the PCC, precuneus, left occipital region, and right frontal region), with the strength of EEG effective connectivity in the delta band being significantly greater than in the other four regions (Figs 3A and 4B). Previous fMRI studies of the DMN have shown that in the eyes-open state the mPFC modulates the activation of the PCC^24^, and that the delta rhythm is closely associated with reward processing and salience detection^55^. Other authors have utilized an EEG source localization approach to confirm that the medial frontal regions are the source of the delta rhythm^56^, supporting our results: in the eyes-open state, the mPFC is regulating brain activity and that the delta rhythm in DMN regions plays important roles.

However, compared to the eyes-closed and eyes-open states, the mind wandering state (the area labeled “W” in Fig. 3A) was found to have the lowest effective connectivity the least for all regions for any frequency band. The effective connectivity between different regions was found to be the lowest significant dDTF value of effective connectivity as represented by the red dots, where the lower the density of red dot clusters in the frequency band areas, the weaker the effective connectivity would be. It would not have a significant influence on the mental state in the neuro-modulatory mechanisms of the DMN. Using an fMRI approach, Fransson *et al*. observed from the effective connectivity analysis that during the mind-wandering state the PCC plays a main regulatory role and communicates with other DMN brain regions^48^. Studies of EEG effective connectivity revealed that when the task difficulty is reduced during the mind-wandering state, the PCC manages the regulation of relevant brain regions^57^, supporting the findings of the present study presented in Fig. 3A. It has been previously proposed that mind-wandering comprises four cognitive states: mind wandering, awareness of mind wandering, shifting of attention, and sustained attention. The four-state process results in cognitive fluctuation^58^, potentially resulting in less effective connectivity compared to eyes-closed and eyes-open states.

### Compared to extroverts, introverts have a stronger modulation capacity of outflow in the PCC and mPFC

Overall, introverts show a stronger outflow modulation of DMN regions operated by both the PCC and mPFC (Fig. 5A,B). Eysenck reports that at rest introverts display a higher inherent cortical arousal level than extroverts^59^. The cortical arousal level may correspond to the degree of mental activity^60^. Several studies have found that higher levels of cortical arousal correspond to increased activity in the PCC^39,61,62^. Scholars believe that the introversion/extroversion personality trait is associated with the DMN^63^, though they rarely investigate changes in the effective connectivity of DMN brain regions with respect to introversion/extroversion. Other authors have reported that introversion/extroversion is associated with exchanges in information flow and effective connectivity between the mPFC and PCC^64^. The PCC was found to be associated with self-centered cognition^61^ and responsible for the integration of information coming from other brain regions^65^, findings consistent with the present results (Fig. 5A). Studies of social cognition have proposed that the mPFC is an important brain region in the mentalizing system^66^. The mentalizing network underlies mental state inference^67^, reflecting the way people interact with their personalities. When an individual considers how to interact with the outside world, the mPFC is activated^20,68^. Other studies have reported that the mPFC and cortical arousal levels are positively correlated^69^, findings consistent with present results (Fig. 5B). Moreover they have shown that the difference between introversion and extroversion is reflected in the spectral power measured in the forebrain and hindbrain^18^. Schmidtke and Heller also detected greater electrical activity in the frontal and posterior areas in extroverts^70^, providing further experimental support to our results (Fig. 2B). These previous studies together with the present results suggest that the PCC and mPFC exert a stronger modulatory action in introverts than in extroverts, and the effect would be enhanced by changes in the cortical arousal level.

In conclusion, the study verified EEG effective connectivity in relevant DMN brain regions of interest (mPFC, PCC, precuneus, the right frontal and the left occipital regions) and the effects of different task-negative conditions (eyes-open/eyes-closed state) or personality trait (introversion/extroversion). Univariate analysis showed that in the eyes-closed state, the PCC was the strongest modulator of outflow through the alpha band, while the mPFC was the strongest modulator in the eyes-open state. Compared with extroversion, introversion exerted a stronger modulation on relevant DMN regions, with the PCC as the prominent modulatory mechanisms of the relevant DMN regions.

## Methods

### Participants

Eighty-five subjects (40 men, 45 women; mean age = 23.5 years) were recruited through online advertisements. The experiment was performed in the Design Perceptual Awareness Lab at the National Taiwan University of Science and Technology (NTUST). The screening criteria were as follows: healthy individuals with no history of mental illness, and no addiction to alcohol, caffeine, or drugs. According to the Declaration of Helsinki^71–73^, the study was approved by the Institutional Review Board of Cathay General Hospital. All methods were carried out in accordance with the approved guidelines. Informed consent was obtained from all subjects prior to the experiments.

### Tasks and procedures

The subjects were presented and asked to read the following instructions for completing the task: “close eyes” for two minutes, “mind wandering” for a minute, and “open eyes” for two minutes (Fig. 1). In the beginning, the computer screen instructed the subject to close their eyes for two minutes. At the end of the two minutes, the computer beeped to remind the subject to open their eyes. Then the screen instructed the subject to allow their “mind to wander” for a minute. One minute later, a beep reminded the subject to open their eyes and look straight ahead at a cross at the center of the screen for two minutes, and finally a beep signaled the end of the experiment. In addition, the subject was requested to stay still. Throughout the experiment, Presentation software (Neurobehavioral Systems, Inc.) was used to control the process and present images on the screen.

The Myers-Briggs Type Indicator (MBTI)^74^ was used to examine the subject’s introversion/extroversion personality traits. After “eyes-closed,” “mind-wandering,” and “eyes-open,” the screen sequentially showed 60 Questions from the MBTI personality test. The subject answered the questions honestly based on how they felt. Each question had seven choices indicating the degree of agreement of the subject, which read from left to the right as very strongly disagree, strongly disagree, disagree, neutral, agree, strongly agree, and very strongly agree, with respective scores of −3, −2, −1, 0, 1, 2, and 3. A compound score greater than 0 indicates an extroverted personality, while a compound score less than 0 indicates an introverted personality. Subjects were asked to choose a single answer for each question. After the test, the subject’s personality trait (introversion/extroversion) was analyzed.

### EEG recording and preprocessing

The EEG signals were acquired using Neuroscan. The Neuroscan software was used to build the EEG database, collect the information contained in the raw EEG data, and analyze and process EEG signals. According to the international 10–10 system of electrode placement, EEG signals at 64-channel electrode positions were collected. The EEG data were collected in the original CNT file format, which was then pre-processed using MATLAB EEGLAB software^75^. In the process of EEG analysis, a wide range of sources of noise was examined to reduce their impact. We used the finite impulse response (FIR) filter^76–78^, followed by a high-pass filter with a cut-off frequency of 1 Hz and a transition band of 0.2 Hz to remove baseline drifting artifacts. Subsequently, a low-pass filter with a cut-off frequency of 50 Hz and a transition band of 7 Hz was adopted to remove muscle artifacts and line noise. The sampling rate of the filtered EEG signals was downsampled to 250 Hz. After noise removal, the clean EEG signals were used for subsequent data analysis^79^.

### Independent component analysis (ICA) and clustering

MATLAB R2013b version was used for the analysis and clustering of EEG data. The Infomax module of EEGLAB was used for the ICA^80–82^ of EEG data collected during the first 5 minutes from the 85 participants (2 minutes of eyes-closed/1 min of mind-wandering/2 minutes of eyes-open). ICA effectively suppresses noise such as eye movement and electrical signals from muscles and can adopt DIPFIT2 routines^83,84^ to calculate the corresponding equivalent dipole location of independent components in the brain. Using the K-means clustering algorithm^85^ including the consistency in the scalp map, the power spectrum density and equivalent dipole location^86^ of 5610 independent components were divided into 12 groups of common brain regions (frontal midline region, left frontal region, right frontal region, central midline region, left temporal region, right temporal region, left motor region, right motor region, parietal region, left occipital region, occipital midline region, and right occipital region - see Fig. 2A). Between 60 and 125 independent components were identified in each brain region. The study detected 95 components in the right frontal region and 99 components in the left occipital regions. The data of the right frontal region and left occipital region were then compared for statistical differences (Wilcoxon rank-sum test, p < 0.05) according to different brain regions and frequencies concerned with task-negative conditions and personality traits as shown in Fig. 2B.

### Measure of effective connectivity (dDTF)

Previously, it was thought that specific brain functions are processed by particular brain regions, known as brain functional localization^87,88^. The brain is an interactive network, as different brain regions are associated with different visual inspections/personality traits states, the interaction between brain regions and the network regulatory mechanisms can be observed. The concept of the DMN has been one of the major topics of neuroscience since its introduction by Raichle in 2001^8^. In addition to the observation of interactions between DMN regions, in the study, brain regions that are associated with eye-opening/eyes-closing and introversion/extroversion were observed. In addition, the interaction between brain regions belonging to the DMN and external to the DMN was examined.

The Source Information Flow Toolbox (SIFT)^44^ of MATLAB was used for the measurement of effective connectivity (i.e., the causal relationship between activity measured in different brain regions) producing Fig. 3. A total of five brain regions were studied: the right frontal region, left occipital region, mPFC, PCC, and precuneus. The DMN regions of interest, the mPFC, PCC, and precuneus, were based on the equivalent dipole location analysis of 5610 ICs (Talairach xyz coordinate values)^32,33^ collected in the present study, which corresponded to the following Brodmann areas^89,90^: the mPFC comprised BA8 (frontal eye fields), BA9 (dorsolateral prefrontal cortex), and BA10 (anterior prefrontal cortex, most rostral part of superior and middle frontal gyri); the PCC comprised BA23 (ventral posterior cingulate cortex) and BA31 (dorsal posterior cingulate cortex); and the precuneus comprised BA7 (visuo-motor coordination). The study detected 273 components in the mPFC, 81 components in the PCC, 74 components in the precuneus, 95 components in the right frontal region, and 99 components in the left occipital regions. The direct Directed Transfer Function (dDTF)^45,46^ was used to calculate the causal relationship between brain regions by calculating the intensity values of effective connectivity between brain regions at different times and EEG frequencies. Figure 3A presents the dDTF value of significant effective connectivity with red dots, the higher the density of red dot clusters in the frequency band areas, the stronger the effective connectivity would be. The dDTF is reported in Eq. (1): the square of dDTF_*ij*_ (f) equals to the square of F_*ij*_ (f) times the square of C_*ij*_ (f), in which dDTF_*ij*_ (f) is between 0 and 1. The greater the value of dDTF returns the level of influence of brain region *i* on the brain region *j* at the frequency *f*, the stronger the causal connectivity between regions *i* and *j*. Fij (f) in Eq. (2) is between 0 and 1, and represents the DTF^45^ between the brain region *i* and brain region *j* calculated at all frequencies. C_*ij*_ (f), between 0 and 1, is the calculated value of partial coherence between the brain region *i* and brain region *j*^91^. H_*ij*_ (f) is the transfer matrix^92^ of the MVAR model between brain region *i* and region *j* at the frequency *f*. The total number of brain regions is denoted as *k* in Eq. (2) for the calculation of the effective connectivity between brain regions.

1 $$dDT{F}_{ij}^{2}(f)={F}_{ij}^{2}(f){C}_{ij}^{2}(f)$$

2 $${F}_{ij}^{2}(f)=\frac{|{H}_{ij}(f){|}^{2}}{{\sum }_{f}\,{\sum }_{m=1}^{k}\,|{H}_{im}(f){|}^{2}}$$

### Information flow analysis

Brain activity is often propagated through information flow from the source to the target brain region^45,93–95^. The dDTF is mainly used for calculating effective connectivity between brain regions; it represents the degree of influence of activity of one brain region on the activity of another brain region. The directional information flow can be used to explain the causal relationship between brain region activity; the inflow represents the sum of dDTF intensity of the influence from all brain regions on a specific region, while the outflow represents the sum of dDTF intensity of the influence of a single brain region on the other brain regions^44,96^. fMRI effective connectivity analysis has been used in previous studies to reveal the interactions between brain regions of the DMN using information flow analysis. It has been shown that effective connectivity has a positive correlation with the blood oxygenation level-dependent (BOLD) signal power^93^.

The effective connectivity matrix of five brain regions (the right frontal region, left occipital region, mPFC, PCC, and precuneus) from 85 subjects was calculated. N × N time-frequency matrices of dDTF were obtained by calculating the subject’s N independent components (ICs) in the five brain regions. The direction of the information flow goes from each column to each row, and each cell along the x axis represents 1–300 seconds, and the y-axis represents 1–70 Hz. The values reported in the matrix represent dDTF values at different times and different frequencies. Each subject’s own time/frequency dDTF matrix can be calculated, yielding a total of 85 different dDTF time/frequency matrices. After the five brain regions were paired, the diagonal was removed and the twenty source-target conditions were computed (e.g. right frontal region [RF] → left occipital region [LO], RF → mPFC, RF → PCC, RF → precuneus, LO → RF, LO → mPFC, LO → PCC, LO → precuneus, mPFC → RF, mPFC → LO, mPFC → PCC, mPFC → precuneus, PCC → RF, PCC → LO, PCC → mPFC, PCC → precuneus, precuneus → RF, precuneus → LO, precuneus → mPFC, and precuneus → PCC). The dDTF values of the time-frequency matrices corresponding to the above twenty brain regions were extracted from a total of 85 dDTF, summed, and averaged to obtain the dDTF time frequency matrix (5 × 5) of the causal relationships of the five brain regions (Fig. 3A). Bootstrap statistics were used by EEGLAB to estimate the differences between conditions (p < 0.05) producing Figs 4 and 5. The processing flow is shown in Fig. 3B. Bootstrapping is a statistical technique that allows means and margins of error for samples and populations that are not normally distributed to be calculated^97,98^.
